# Characterization of Conformation-Sensitive Antibodies to ADAMTS13, the von Willebrand Cleavage Protease

**DOI:** 10.1371/journal.pone.0006506

**Published:** 2009-08-05

**Authors:** Zuben E. Sauna, Chinyere Okunji, Ryan C. Hunt, Tanvi Gupta, Courtni E. Allen, Elizabeth Plum, Adam Blaisdell, Vahan Grigoryan, Geetha S, Robert Fathke, Kenji Soejima, Chava Kimchi-Sarfaty

**Affiliations:** 1 Laboratory of Hemostasis, Division of Hematology, Center for Biologics Evaluation and Research, Food and Drug Administration, Bethesda, Maryland, United States of America; 2 National Center for Biotechnology Information, National Library of Medicine, National Institutes of Health, Bethesda, Maryland, United States of America; 3 First Research Department, the Chemo-Sero-Therapeutic Research Institute, Kumamoto, Japan; The Scripps Research Institute, United States of America

## Abstract

**Background:**

The zinc metalloprotease ADAMTS13 is a multidomain protein that cleaves von Willebrand Factor (VWF) and is implicated in Thrombotic Thrombocytopenic Purpura (TTP) pathogenesis. Understanding the mechanism of this protein is an important goal. Conformation sensitive antibodies have been used to monitor protein conformation and to decipher the molecular mechanism of proteins as well as to distinguish functional and non-functional mutants.

**Methodology/Principal Findings:**

We have characterized several antibodies against ADAMTS13, both monoclonal and polyclonal. We have used flow cytometry to estimate the binding of these antibodies to ADAMTS13 and demonstrate that antibodies raised against the TSP and disintegrin domains detect conformation changes in the ADAMTS13. Thus for example, increased binding of these antibodies was detected in the presence of the substrate (VWF), mainly at 37°C and not at 4°C. These antibodies could also detect differences between wild-type ADAMTS13 and the catalytically deficient mutant (P475S). The flow cytometry approach also allows us to estimate the reactivity of the antibody as well as its apparent affinity.

**Conclusions/Significance:**

Our results suggest that these antibodies may serve as useful reagents to distinguish functional and non-functional ADAMTS13 and analyze conformational transitions to understand the catalytic mechanism.

## Introduction

ADAMTS13 is a protease that cleaves the von Willebrand Factor (VWF) within intact blood vessels under shear stress [1]–[3]. VWF is a large glycoprotein secreted by vascular endothelial cells as multimers. At a region of vascular injury, the multimeric form of VWF initiates the clotting process by adhering to platelets. A reduction or elimination of the protease activity of ADAMTS13 results in the VWF multimers remaining uncleaved in the circulating blood stream, which ultimately leads to intravascular thrombosis and an associated disorder known as Thrombotic Thrombocytopenic Purpura (TTP) [4], [5]. Thus ADAMTS13 plays a critical role in maintaining intravascular homeostasis.

ADAMTS13 is a member of the ADAMTS (a disintegrin and metalloproteinase with thrombospondin motifs) family of proteins [6] and is secreted by almost all tissues but primarily by hepatic stellate cells [7]–[10]. The ADAMTS family of proteins are secreted metalloproteases characterized by multiple domains [11]. The domain structure of ADAMTS13 contains a signal peptide, propeptide, a metalloprotease, disintegrin, a thrombospondin type 1 domain (TSP1), a cystein rich domain, a spacer domain, seven TSP1 repeats and two C-terminal CUB (C1r/C1s, Urinary EGFand Bone morphogenetic protein) domains. Although all ADAMTS proteins have characteristic multi domains, the ADAMTS13 is unique in having two additional C-Terminal CUB domains and an unusually short propeptide. Moreover, unlike other ADAMTS members, ADAMTS13 is catalytically active prior to secretion from the cells [12]. The metalloprotease domain is the catalytic domain and responsible for the protease activity [13]. The region from the disintegrin to the spacer domains is involved in substrate recognition [14] while the distal C-terminal TSP1 repeats and CUB domains are also necessary for its activity under flowing conditions [15], [16]. The role of the cysteine-rich domain is more controversial [14], [17]. It is thus evident that the ADAMTS13-VWF interaction is complex; several domains of the protein are involved in the regulation of VWF cleavage.

Antibodies sensitive to the conformation of a protein have been successfully used to understand the structural organization of proteins, distinguish their functional and non-functional forms, elucidate molecular mechanisms, and establish the role of different domains of multidomain proteins. For example, conformation-sensitive antibodies against native and denatured bovine somatotropin have been used successfully to study its folding, stability, thermal denaturation and refolding [18]. The specific monoclonal antibody 5D3 was used to differentiate functional and non-functional ABCG2 proteins which had functional ATP- and drug-substrate-binding sites but differed in the formation of a catalytic intermediate [19]. A monoclonal antibody against the CRIB domain of the N-WASP protein that specifically recognized the activated protein was used to localize it within cells [20]. Conformation-sensitive UIC2 could identify different classes of drug modulators of P-glycoprotein and the molecular mechanism underlying their interactions based on mutations in the multidrug resistance gene (*MDR*1; *ABCB*1) which led to different conformations of the molecule [21], [22]. Similarly, a conformation-sensitive antibody was successfully used to identify the disease-causing mutation in Copper/Zinc Superoxide Dismutase [23].

ADAMTS13 is a multi-domain protein with complex interactions with its substrate. Antibodies sensitive to different conformations could thus be potentially useful to understand the regulation and mechanism of ADAMTS13-mediated catalysis. In this report, we studied and defined new conformation-sensitive antibodies to ADAMTS13. We examined the monoclonal antibodies Wh2-11-1 and Wh2-22-1A, which are specific to the TSP1-4 and Disintegrin domains, respectively, and the polyclonal antibody BL154G that is specific to the metalloprotease domain of ADAMTS13, for their sensitivity to conformation. Here we show their ability to detect conformation changes in the ADAMTS13 protein under various conditions in which the protein normally undergoes changes in conformation such as changes due to temperature, and in the presence of its substrate. We use flow cytometry to measure the affinities and reactivity of these antibodies. We demonstrate that both the reactivity and the apparent affinity (concentration required for half-maximal binding) of these antibodies is altered by the addition of substrate (VWF). This change is observed mainly at 37°C but not at 4°C and occurs in the wild-type protein but not in a catalytically inactive mutant. These results indicate that the ADAMTS13 antibodies are conformation-sensitive.

## Results

### Using antibodies against ADAMTS13 in flow cytometric assays

**Figure 1:**
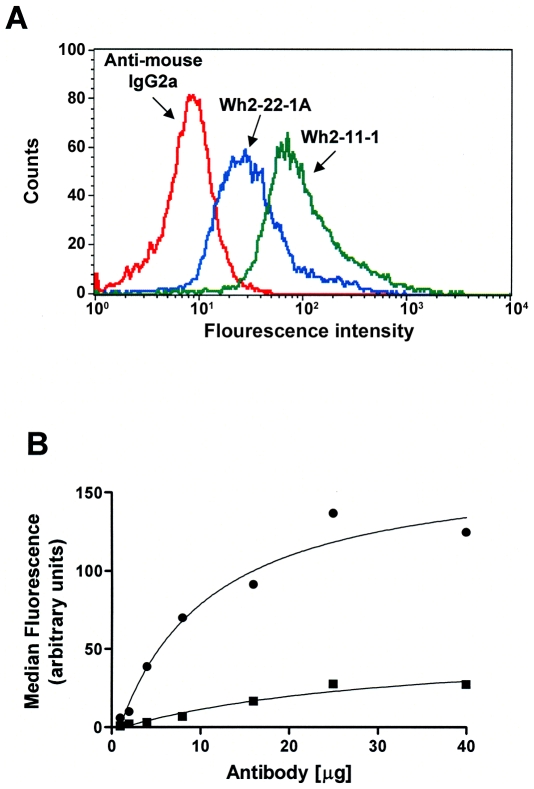
Flow cytometry based assay to detect intracellular ADAMTS13. (A) HEK293 cells transiently transfected with ADAMTS13 plasmid DNA were permeabilized and incubated with the anti ADAMTS13 antibodies Wh2-22-1A or Wh2-11-1 and an isotypic control antibody Anti-mouse IgG2a. The cells were washed to remove excess antibody and treated with an Alexa Flour 488 labeled secondary antibody. The fluorescence associated with the Alexa Flour 488 was measured in a flow cytometer and histograms showing the distribution of fluorescence intensity in a population of 10,000 cells following the different treatments are depicted. Cells treated with the control antibody show low levels of fluorescence that establishes the baseline for nonspecific interactions with the control and secondary antibodies. Histograms show permeabilized HEK293 cells incubated with the isotypic control antibody (red line), Wh2-22-1A (blue line) or Wh2-11-1 (green line) prior to incubation with the secondary antibody. (B) Apparent affinity and reactivity of the anti-ADAMTS13 mAbs Wh2-22-1A (•) and Wh2-11-1 (▪) was estimated using the flow cytometry based assay depicted in (A) above. The cells were incubated with increasing amounts of the antibodies and the median fluorescence (of histograms similar to those depicted in (A) above) were plotted as a function of µg antibody.

Monitoring the antigen-antibody interaction of a protein in an intact cell using flow cytometry has the significant advantage that the antibody can explore the tertiary structure of a functional protein in its native environment. In addition, it is plausible that some antibodies bind to epitopes which are sensitive to the conformation of the protein which may have functional or diagnostic importance. Soejima and coworkers have characterized antibodies that recognize several distinct domains of ADAMTS13; nonetheless the antibodies were raised by inoculating mice with the intact protein and not peptide fragments (Soejima, personal communication). In Fig. 1A we demonstrate that the antibodies Wh2-22-1A (that recognizes the disintegrin-like domain) and Wh-2-11-1 (that recognizes the thrombospondin type 1–4 domains) bind to intracellular ADAMTS13. Human embryonic kidney (HEK293) cells were transiently transfected with ADAMTS13 plasmid DNA as described in the Materials and methods and expression of ADAMTS13 in this system has previously been shown to be equivalent to that in human stellate liver cells, which are known to express ADAMTS13 at high levels [25]. The cells were permeabilized and incubated with the anti ADAMTS13 antibodies and washed to remove excess antibody that did not react with the antigen. The cells were then incubated with a secondary antibody tagged with Alexa Flour 488 (see Materials and methods for details). The control cells were incubated with the isotype IgG2a antibody followed by incubation with the secondary antibody. The fluorescence associated with the Alexa Flour 488 was measured in a flow cytometer and Fig. 1A shows the distribution of fluorescence intensity in a population of 10,000 cells following the different treatments. Cells treated with the IgG2a control antibody show low levels of fluorescence that establishes the baseline for nonspecific interactions and interactions with the secondary antibody alone. However, when the cells were treated with Wh2-11-1 or Wh2-22-1A antibodies prior to addition of the secondary antibody there is a dramatic increase in the fluorescence from a median fluorescence value of 23.5 to 165.48 or 67.32. It is thus clear that these antibodies which have previously been shown to recognize ADAMTS13 in an immunoblot assay also bind to intracellular recombinant ADAMTS13 and flow cytometry can be used to characterize this interaction (Geetha S *et al* unpublished results).

### Dose-dependent binding of ADAMTS13-specific monoclonal antibodies

In the previous section we have demonstrated that a flow cytometric assay can be used to quantitatively assess the binding of antibodies against ADAMTS13 to the intracellular protein. These experiments demonstrate that by permeabilizing cells it is possible for anti-ADAMTS13 antibodies to access the interior of cells as well as bind to the intracellular ADAMTS13. Thus this technique can potentially be used to study the dose-dependent saturating binding of the ADAMTS13-specific monoclonal antibodies. We therefore used the flow cytometry-based assay to estimate the concentration required for half-maximal binding of the monoclonal antibodies Wh2-11-1 and Wh2-22-1A to ADAMTS13 which is a measure of the apparent affinity of the antibody for the protein (Fig. 1B). Our results show that although both antibodies bind specifically to ADAMTS13 (Fig. 1A) the concentration of antibodies required for half-maximal binding as well as the V_max_ are very different. This is not surprising as these antibodies recognize different epitopes on ADAMTS13. Moreover, the importance of estimating the reactivity of antibodies that may be sensitive to the conformation of the protein is that such antibodies would bind with increased or decreased efficiency when the conformation of the epitope is altered.

### The mAb Wh2-11-1 shows increased reactivity to ADAMTS13 in the presence of substrate (VWF)

**Figure 2:**
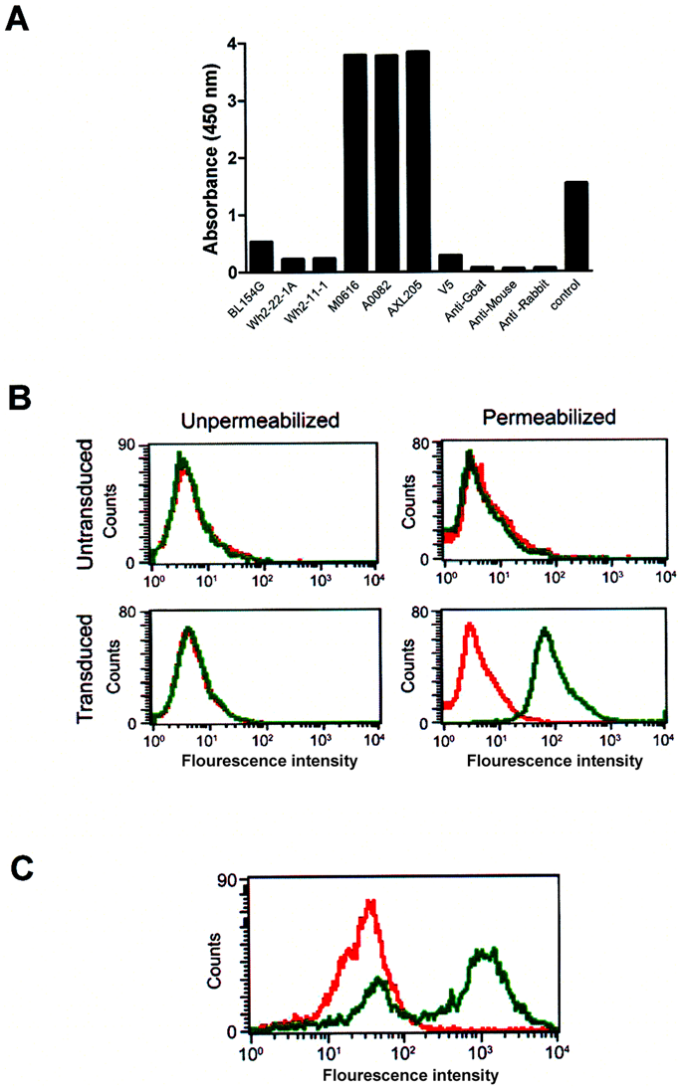
Identification of intracellular VWF. (A) ELISA assay showing reactivity of different antibodies to VWF. VWF was allowed to bind collagen coated wells and antibodies raised against ADAMTS13 (BL154G, Wh2-22-1A and Wh2-11-1) [35], VWF (MO616, A0082 and AXL205) or the anti-V5 (V5) as well as secondary antibodies (all HRP conjugated - anti-goat, anti-mouse or anti-rabbit) were added to the wells to measure their reactivity. The positive control was an antibody against VWF provided by the manufacturer of the kit. The antibodies were used at a dilution of 1∶1000, in plates coated with VWF. (B) Control HEK293 cells and those transiently transfected with VWF were analyzed by flow cytometry for intracellular VWF. Cells were treated with either secondary antibody alone or the anti-WVF monoclonal antibody AXL205 followed by the secondary antibody. The left panel depicts cells that were unpermeabilized while the right panel shows cells that were permeabilized prior to treatment with the antibodies. The histograms show cells treated with the secondary antibody alone (red line) or the anti-VWF antibody followed by the secondary antibody (green line). (C) The ADAMTS13-transduced HEK293 was harvested 24 hours post-transduction and 500 µl of VWF was added to these cells followed by incubation at 37°C for 10 min. The cells were then immunostained with AXL205 and the appropriate secondary antibody and analyzed by flow cytometry. The histograms show cells stained with the secondary antibody alone (red line) or the primary antibody followed by secondary antibody (green line).

Enzymes undergo conformational changes during the catalytic process and conformation sensitive antibodies offer a means of monitoring these changes. The substrate for ADAMTS13 is a large, multimeric glycoprotein VWF (the monomeric unit is 260 kDa, and the multimer is greater than 10,000 kDa) and it would be useful to establish whether the binding of VWF affects the kinetic parameters (apparent K_d_ or V_max_) associated with the binding of the monoclonal antibodies to intracellular ADAMTS13. Such a measurement assumes that there is no interaction between the anti-ADAMTS13 antibody and VWF. As this is an important control we tested antibodies raised against ADAMTS13 as well as against VWF in an ELISA assay using VWF bound to collagen coated wells. Our results show that none of the anti-ADAMTS13 antibodies tested interacts with the VWF; on the other hand three different anti-VWF antibodies (which provide a positive control) show a strong signal (Fig. 2A). Secondly the fact that VWF is a large multimeric protein raises the concern that VWF may not enter cells, even those that have been permeabilized. We used a flow cytometric assay similar to that described in Fig. 1A to monitor VWF which is added to permeabilized cells and incubated for 10 minutes at 37°C. Fig. 2B shows that HEK293 cells transiently transfected with VWF show a higher fluorescence signal (associated with the binding of the anti-VWF monoclonal antibody AXL205) than the untransfected cells. However addition of extrinsic VWF to the untransfected control cells results in the intracellular accumulation of VWF (Fig. 2C) as evidenced by the fact that the fluorescence signal following incubation with the anti-VWF antibody is at a level comparable to that observed in cells transfected with VWF. However, when the same experiment was performed with unpermeabilized cells, the histograms with the secondary antibody alone and the anti-VWF monoclonal antibody AXL205 could not be distinguished (Data not given), suggesting that we are detecting VWF that has entered the cell and not merely adsorbed on the cell surface. Taken together, these results clearly show: (i) Permeabliziation of cells permit the entry of large molecules such as antibodies and VWF. (ii) Anti-ADAMTS13 and anti-VWF antibodies can be used (in conjugation with the appropriate fluorescence tagged secondary antibody) to detect intracellular levels of the two proteins. (iii) Flow cyotmetry offers a means of quantifying intracellular levels of ADAMTS13 and VWF. Our experimental system can, therefore, be used to study the effect of VWF binding to ADAMTS13 as estimated by changes in the dose-dependent binding of conformation sensitive antibodies to ADAMTS13.

**Figure 3:**
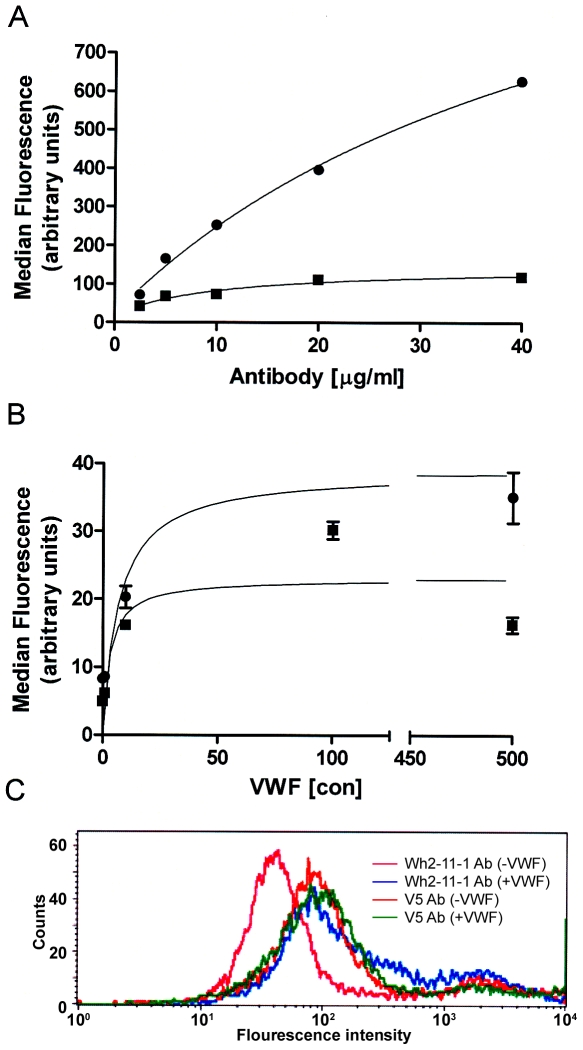
VWF increases the immunoreactivity of anti-ADAMTS13 antibody Wh2-11-1. (A) Serial dilutions of the mAb Wh2-11-1 were allowed to bind to ADAMTS13 from human embryonic cells (HEK293 cells) in the absence (▪) or presence (•) of 100-500 µl from VWF stock. (B) Either 1 µg (▪) or 5 µg (•) of the mAb Wh2-11-1 were allowed to bind ADAMTS13 in the presence of increasing concentrations of VWF. The binding of the antibody in both experiments was monitored using flow cytometry and the data plotted using the curve-fitting software Graphpad Prism. (C) The binding of the mAb Wh2-11-1 was compared to that of the anti-V5 antibody in the absence or presence of VWF using flow cytometry. The histograms represent: Wh2-11-1 alone (red), Wh2-11-1 in the presence of VWF (green), anti-V5 alone (pink) and anti-V5 in the presence of VWF (blue). The figures represent data from one of three independent experiments.

We analyzed the effect of the ADAMTS13 substrate, VWF, on the reactivity of the mAb Wh2-11-1. Incubation of VWF with ADAMTS13 at 37°C, resulted in a >10-fold increase in the reactivity of Wh2-11-1 (P<0.001). Moreover, this increase in reactivity is accompanied by a ∼5.5-fold decrease in the apparent affinity of the mAb for ADAMTS13 (concentration required for half-maximal binding increases from 9.7 µg/ml to 50.6 µg/ml; Fig. 3A). These results indicate that binding of VWF alters the conformation of the ADAMTS13 epitope resulting in a decreased apparent affinity of the mAb Wh2-11-1. The presence of the substrate may be expected to protect some binding sites on ADAMTS13 and if this were the case, one may expect a decrease in the reactivity of the mAb. However, Fig. 3B shows that the increased reactivity of the mAb Wh2-11-1 in the presence of VWF is concentration dependent and saturable. The fact that there is a large increase in the reactivity of the antibody strongly indicates that the mAb is sensitive to conformational changes in ADAMTS13. Furthermore, we have shown (Fig. 2A) that all three tested ADAMTS13 antibodies do not bind to VWF and therefore the increase in binding in the presence of VWF results only from specific binding to ADAMTS13. We also demonstrate (Fig. 3C) that while the mAb Wh-2-11-1 shows an increase in reactivity on the binding of substrate (VWF) in cells transfected with rADAMTS13, an antibody to the V5 tag on rADAMTS13 (which is not sensitive to the conformation of the epitope) shows negligible change.

**Figure 4:**
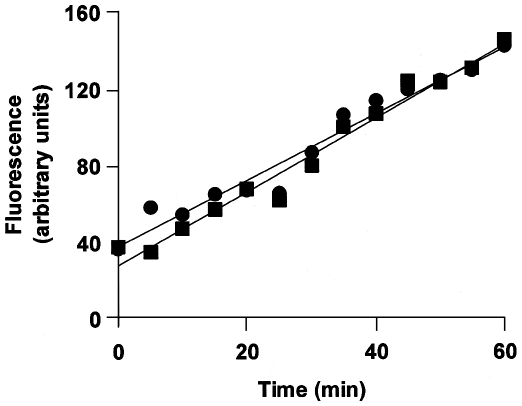
Permeabilization of cells does not affect the activity of intracellular ADAMTS13. Activity of ADAMTS13 in control cells (•) and those treated with the permeabilization reagent (IntraPrep^TM^ Beckman Coulter) (▪) was measured using the FRETS-VWF73 assay (for details of permeabilization and the FRETS-VWF73 assay see Materials and methods). Following treatment the cells were lyzed and the cytoplasmic fraction was concentrated and equal volumes were used from control and permeabilized cells in the FRET-based assay. The amount of total protein was subsequently estimated and equal amounts of protein were loaded for an immunoblot using the anti-V5 antibody. Relative amounts of ADAMTS13 were estimated from the immunoblot and the data presented here are corrected based on these values. The figure represents data from three independent experiments.

We have suggested, above, that the changes in ADAMTS13 within cells detected by the conformation sensitive antibodies may be related to catalytic activity. This proposition is based, in part, on the observation that an increased (and concentration dependent) binding of antibodies occurs in the presence of the substrate, WVF. The presentation of the large VWF to the intracellular ADAMTS13 however requires that the cells be permeabilized. It is therefore important to demonstrate that permeabilization *per se* does not affect the activity of ADAMTS13. ADAMTS13-mediated cleavage of the synthetic 73-amino-acid peptide, FRETS*-*VWF73 can be detected using fluorescence resonance energy transfer and this assay has increasingly been used to quantify ADAMTS13 activity [24]. We used this assay to measure the initial rates of intracellular ADAMTS13 proteolytic activity in control cells and those that had been permeabilized as described in the Materials and methods. Our results show that the activity of intracellular ADAMTS13 is indistinguishable in untreatted cells and those that were permeabilized (Fig. 4).

Taken together, these data suggest that the interaction of VWF and ADAMTS13 results in increased accessibility of the Wh2-11-1 epitope. The Wh2-11-1 epitope is TSP1-4 (thrombospondin type-1-4 motif) and TSP1 repeats of ADAMTS family members have been shown to be important for substrate recognition and cleavage [16].

### The reactivity of mAb Wh2-11-1 is sensitive to temperature

**Figure 5:**
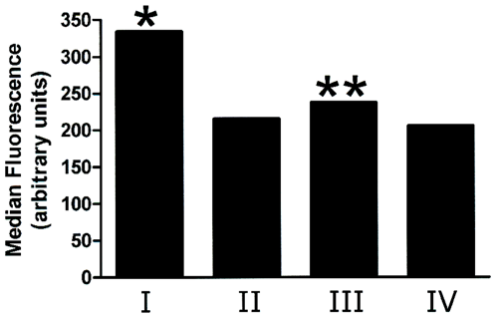
VWF induces conformational changes in ADAMTS13 at 37°C which are inhibited by prior labeling with the mAb Wh2-11-1. ADAMTS13 from HEK293 cells was incubated with the mAb Wh2-11-1 and VWF under the following conditions: (I) ADAMTS13 was treated with VWF at 37°C prior to addition of Wh2-11-1. (II) ADAMTS13 was treated with Wh2-11-1 at 37°C followed by the addition of VWF. (III) ADAMTS13 was treated with VWF at 4°C prior to addition of Wh2-11-1. (IV) ADAMTS13 was treated with Wh2-11-1 at 4°C followed by the addition of VWF. *Reactivity of ADAMTS13 to Wh2-11-1 is significantly higher (p<.001) in the presence of VWF at 37°C. **There is no significant difference between the reactivity of Wh2-11-1 to ADAMTS13 in the presence or absence of VWF at 4°C (p = 0.083). Binding of mAb was monitored by flow cytometry. The figure represents data from one of five independent experiments.

Conformational changes in proteins (particularly those that accompany enzyme catalysis) are often energy dependent; thus, we monitored the reactivity of the mAb Wh2-11-1 to ADAMTS13 at 4°C and 37°C in the presence of the substrate VWF (Fig. 5). Incubation of ADAMTS13 with VWF at 37°C prior to addition of the mAb resulted in increased reactivity (Bar I, Fig. 5). However, when the mAb was added prior to VWF there was reduced reactivity with the mAb (Bar II, Fig. 5), suggesting that binding of the mAb hinders the conformational changes that accompany ADAMTS13-mediated proteolysis. This is consistent with the finding that autoantibodies that recognize various domains of ADAMTS13 (including those similar to the Wh2-11-1 epitope) result in acquired TTP [26] and the fact that the immunogenicity of clotting factors such as factor VIII is a significant impediment to the treatment of hemophilia [27]. This observation also provides additional evidence that Wh2-11-1 interacts with the functional intracellular ADAMTS13 [12]. Finally, addition of VWF at 4°C, either before or after addition of the mAb, does not affect the reactivity of the mAb (Bars III and IV, Figure 3). It would be expected that like most proteolytic enzymes, the optimal activity of ADAMTS13 cleavage occurs between 30°C and 37°C [28] and there is minimal activity at 4°C. Thus, it would be unlikely that VWF-induced conformational changes would occur at 4°C. The results depicted in Fig. 5 suggest that the changes in the reactivity of the mAb Wh2-11-1 reflect conformational changes that accompany catalysis.

### Binding of the anti-ADAMTS13 antibody BL154G prevents substrate-induced conformational changes

**Figure 6:**
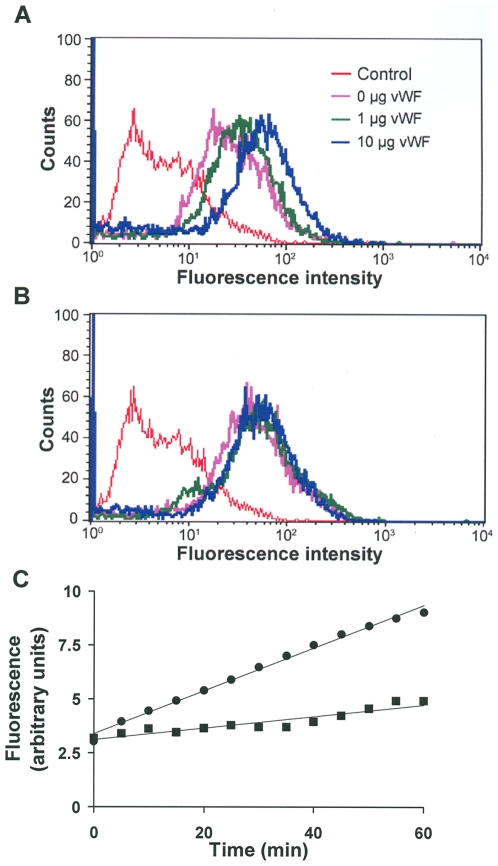
The pAb BL154G inhibits VWF-induced conformational changes in ADAMTS13. (A) ADAMTS13 was incubated with increasing concentrations of VWF for 30 min at 37°C followed by the addition of the pAb BL154G. (B) ADAMTS13 was incubated with BL154G antibody for 30 min at 37°C. Increasing concentrations of VWF were then added to the samples. Both sets of samples were analyzed by flow cytometry. Histograms represent binding of mAb in the presence of 0 (red), 100 (purple), 250 (green) and 500 (blue) µl of VWF stock. The figures represent data from one of three independent experiments. (C) Activity of ADAMTS13 in the absence (•) and presence (▪) of the pAb, BL154G, measured using the FRETS-VWF73 assay.

The data presented above shows that the monoclonal antibody Wh2-11-1 can be used to report conformational changes in ADAMTS13 such as those resulting from interactions with the substrate. Several reports have indicated that autoantibodies against ADAMTS13 inhibit this protease [5], [29]–[33]. We therefore studied the effect of the polyclonal antibody BL154G on conformation changes in ADAMTS13 as well as its function. In Fig. 6A we show that addition of VWF increases the reactivity of the polyclonal Ab BL154G in a concentration dependent manner, similar to our observation with Wh2-11-1. In this experiment permeabilized cells transfected with an ADAMTS13 encoding vector were incubated with increasing concentrations of VWF for 10 min at 37°C. Cells were then exposed to the polyclonal antibody BL154G for 30 min, cells were washed and incubated with the secondary antibody tagged with (FITC rabbit anti-goat). The ADAMTS13 transduced cells show a higher fluorescence signal than the control (untransfected) cells and the fluorescence signal is enhanced in a concentration-dependent manner with the addition of increasing amounts of VWF. However, when ADAMTS13 was treated with the antibody prior to the addition of VWF, there was no change in reactivity (Fig. 6B). Thus, binding of the polyclonal antibody appears to inhibit conformational changes in the protease. It is plausible that these conformational changes are associated with catalysis and could reflect the functional state of the ADAMTS13 protein. We used the FRET-based assay described above to monitor the activity of ADAMTS13 activity before and after treatment with the antibody, BL154G. The data depicted in Fig. 6C clearly shows that pre-treatment of ADAMTS13 with BL154G significantly inhibits catalytic activity.

### Intracellular and secreted forms of ADAMTS13 show comparable proteolytic activity

**Figure 7:**
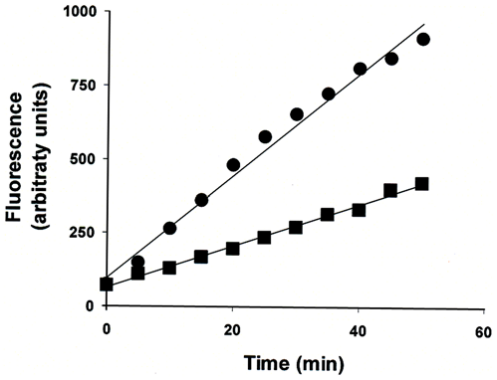
The activity is secreted and intracellular ADAMTS13. Activity of secreted (•) and intracellular ADAMTS13 (▪). Activity of intracellular ADAMTS13 was measured using the FRETS-VWF73 assay described in Fig. 4. The secreted ADAMTS13 was collected from the media which was concentrated using a Centriprep 30 centrifrugal filter device. The data were corrected for amount of ADAMTS13 in each sample as described in Fig. 4. The activities for the secreted and intracellular ADAMTS13 were 17.3 arbitrary units/min/unit protein and 8.8 arbitrary units/min/unit protein respectively. Typical traces from three independent experiments are depicted.

We have shown above that monoclonal antibodies sensitive to protein conformation may be useful in the repertoire of tools used to study ADAMTS13. One drawback of the flow cytometry based assay described here is that it can only be performed in intact cells. We have therefore studied the conformation changes in intracellular ADAMTS13. It is thus important to demonstrate that intracellular ADAMTS13 shows comparable activity to the secreted form which is the physiologically relevant form of the protein. An earlier study has demonstrated that the intracellular, pro-ADAMTS13, cleaves the VWF factor intracellularly [12]. This observation was based on a qualitative assay where proteolytic cleavage of VWF was determined on an immunoblot and has not been independently verified. We have used the FRET based assay described in Fig. 6C to compare the activities of the intracellular and secreted forms of ADAMTS13 (Fig. 7). The results depicted are corrected for the relative amounts of intracellular and secreted forms of ADAMTS13 detected on an immunoblot when equal amounts of total protein were loaded on the gel. Our results clearly show that consistent with the previous study [12] both secreted and intracellular ADAMTS13 exhibit proteolytic activity. However using the FRET based assay which permits better quantification, it is possible to estimate that the initial rates are slightly higher in the secreted ADAMTS13 (Fig. 7).

### A catalysis-deficient mutant of ADAMTS13 does not show VWF-induced changes in conformation

**Figure 8:**
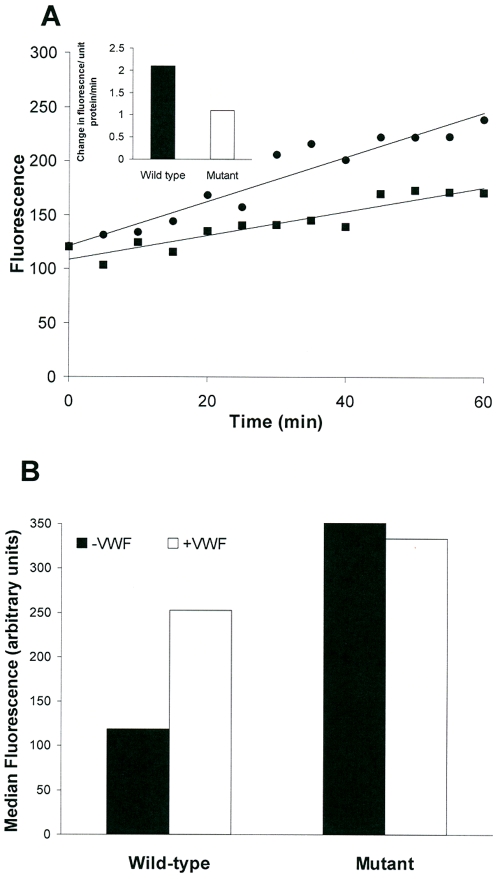
A functionally deficient mutant of ADAMTS13 shows minimal conformation change in the presence of VWF. (A) Activity of wild-type (▪) and the P475S mutant (•) ADAMTS13s were measured by the FRET-VWF73 assay as described previously. Inset shows the activity of wild-type (filled bar) and the mutant (empty bar) ADAMTS13. (B) Wild-type and the P475S mutant ADAMTS13 were incubated with either BL154G alone (filled bars) or in the presence of 500 µl of VWF stock (empty bars). The VWF was incubated with ADAMTS13 prior to addition of the polyclonal Ab. Binding of polyclonal Ab was monitored by flow cytometry. The figures represent data from one of three independent experiments.

Fujimura and coworkers have studied the effects of common mutations and polymorphisms in ADAMTS13 on protease activity [34]. They demonstrate that a mutant ADAMTS13 (P475S) shows similar expression to the wild type protein but reduced activity. They estimated the activity based on the accumulation of monomers and rate of reduction in multimeric level as identified by sodium dodecyl sulphate-agarose gel electrophoresis. Using the more quantitative FRET based assay for ADAMTS13 activity described in the previous section we confirmed these results (Fig. 8A). Our results show a clear decrease in the rate of FRET-VWF73 cleavage in mutant enzyme compared to the wild type. Even more significant is our observation that the change in conformation of ADAMTS13 on binding of VWF reported by the antibody BL154G does not occur in the mutant protein. We show that incubation of wild-type ADAMTS13 with VWF followed by addition of the antibody BL154G results in an increase in the median fluorescence in a flow cytometry assay (Fig. 8B, bars II & II). However when the experiment is conducted using the mutant ADAMTS13, we did not observe any change in the median fluorescence on addition of VWF. Similar results were obtained with the mAb Wh-2-11-1, where the fluorescence intensity increased 3.5 fold for the wild-type ADANTS13, but only 1.9 fold for the mutant (data not given). Considering that the wild-type and mutant express at equivalent levels (see above) and that the wild-type and mutant proteases show different reactivity to the antibodies suggests different conformations for these forms. Also, while addition of VWF to wild-type ADAMTS13 results in increased reactivity of both the mAb and the pAb, a similar increase in the reactivity of the mAb and the polyclonal Ab is not observed in the mutant ADAMTS13. These results provide further evidence that only the functional ADAMTS13 undergoes conformational changes on interacting with the VWF.

## Discussion

Soejima and coworkers have recently generated several monoclonal antibodies that recognize distinct functional regions of the ADAMTS13 molecule [35]. We found that several of these are sensitive to changes in the conformation of ADAMTS13. Here we characterize in detail three antibodies (the monoclonal antibodies Wh2-11-1 and Wh2-22-1A as well as the polyclonal antibody BL154G). We have used flow cytometry to study the interactions between the antibody and ADAMTS13 so that the native conformation of the ADAMTS13 protein could be preserved. The apparent affinities of all three antibodies were altered in the presence of the substrate (VWF). These antibodies also showed different reactivity to ADAMTS13 in the presence of VWF, but only at 37°C and not at 4°C. Furthermore if the antibodies were added to the ADAMTS13 prior to the addition of VWF there was no change in the reactivity. Addition of VWF to intracellular ADAMTS13 required the permeabilization of cells, however the procedure had no effect on ADAMTS13 activity. These results suggested that these antibodies (and particularly Wh2-11-1) may be useful as conformation-sensitive antibodies to understand the mechanism of ADAMTS13.

Earlier work has shown that Wh2-11-1 recognizes an epitope that corresponds to the TSP1-4 motifs [35] which do not directly interact with the substrate (VWF), but was shown under shear stress to take part in the recognition of the substrate. Consistent with this view is our observation that VWF does not inhibit binding of Wh2-11-1 to ADAMTS13. In this study we show concentration-dependent reactivity of Wh2-11-1 to ADAMTS13 and demonstrate that the addition of VWF results in a dramatic increase in reactivity and a smaller decrease in the apparent affinity of the antibody for ADAMTS13. As there is an approximately 8-fold increase in the immunoreactivity, it is unlikely that the change in reactivity is a consequence of competition between VWF and Wh2-11-1. We also show in this study that the VWF-induced increase in the reactivity of the antibody occurs at 37°C but not at 4°C (Fig. 3). Also significant is the fact that pre-treatment of ADAMTS13 with Wh2-11-1 prior to addition of VWF does not result in an increase in the immunoreactivity. Taken together, these results suggest that although the TSP1-4 domains may not interact directly with the VWF, the consequence of VWF binding is a conformation change (possibly transient) that results in a greater exposure of the Wh2-11-1 epitope, which might mimic shear stress conditions. In addition, the fact that Wh2-11-1 binds to its epitope with different affinities in the presence and absence of VWF suggests that the conformation of this epitope is altered, which might occur in these circumstances. This hypothesis is moreover supported by the fact that the conformational change is energetically driven (i.e. does not occur at 4°C). These results suggest that Wh2-11-1 preferentially binds to a transient conformation during the enzymatic hydrolysis of VWF.

Conformational transitions almost always accompany enzymatic activity, protein-protein interactions or transport mediated by membrane proteins [36]. The ability to track these transitions experimentally has often proved useful in understanding molecular mechanisms. These conformational changes are often identified by fluorescence, EPR or other probes crosslinked to the proteins being studied [37], [38]. This crosslinking approach has two significant disadvantages. Perturbations related to the chemical modifications of the protein often affect the function and/or kinetics of the enzyme or transporter [39]. Secondly, it is extremely difficult to localize probes in the functional domain of the protein. Here again, a common experimental technique involves the replacement of native cysteines in the protein of interest and introduction of cysteines at desired positions followed by chemical cross-linking using thiol-reactive probes [37]-[40]. Such an approach involves the use of mutants as well as chemical cross-linking. A relatively non-invasive means of following functional transitions, on the other hand, is the use of conformation-sensitive antibodies. There are several examples in the literature of the usefulness of this approach [18]–[21], [41]. Extensive characterization of the ADAMTS13 antibody Wh2-11-1 demonstrates that the immunoreactivity of this antibody is sensitive to the conformation of ADAMTS13. Our results also show that the reactivity of this antibody is sensitive to the binding of VWF (which is the substrate of ADAMTS13 for proteolytic degradation). Thus we demonstrate that the interaction of VWF with ADAMTS13 brings about changes in conformation at the TSP1-4 domains (where no direct interactions have been shown to occur). This view is supported by emerging concepts about the role of the ADAMTS13 domains [16], [42]. We also show that a conformation-sensitive antibody can distinguish between the wild-type ADAMTS13 and the mutant P475S. This mutant has been shown previously to have reduced enzymatic activity [34] and we have confirmed in this study that the mutant has decreased hydrolytic activity for FRET-VWF73 (a fluorogenic substrate of ADAMTS13). Finally, the large (∼8-fold) increase in the reactivity of Wh2-11-1 in the presence of VWF is not observed in the mutant ADAMTS13. This observation suggests that the conformational change reported by Wh2-11-1 is not an artifact but represents a reaction intermediate during the ADAMTS13-mediated proteolysis of VWF. A significant underlying assumption of these proposals is that the intracellular and secreted ADAMTS13 (which is physiologically relevant) both exhibit proteolytic activities. A previous study concluded that this was indeed so [12], we confirm this observation in our system in the current study using a more quantitative assay than was used previously. We suggest that the reactivity of the antibodies Wh2-11-1, BL154G and Wh2-22-1A (particularly Wh2-11-1) can be used as a sensitive assay to distinguish functional and non-functional ADAMTS13 as well as to analyze conformational transitions associated with function. For example a recent study determined that several Swedish families with a synonymous mutation (i.e. one with no change in the amino acid sequence) in the Factor IX gene nonetheless exhibit hemophilia B [43]. The authors were however unable to provide a mechanistic explanation for this important observation. We have demonstrated previously that even silent mutations can affect protein folding and function by altering the rate of protein synthesis [44]. Antibodies sensitive to protein conformation are an invaluable tool in testing such hypotheses.

## Materials and Methods

### Cell culture and cell lines

The ADAMTS13-expressing kidney and liver cell lines HEK293 (human embryonic kidney cells; American Type Culture Collection Manassas, VA), and LX2 (human stellate liver cells; a gift from Dr. Friedman, Mt. Sinai, NY) were maintained in DMEM media (Dulbecco's Modified Eagle Medium) with 10% fetal bovine serum (FBS), 50 mg/mL penicillin, 50 mg/mL streptomycin and 5 mM L-glutamine (all obtained from Invitrogen™, Carlsbad, CA). Cells were incubated at 37°C, in 5% CO_2_ with humidity.

### Site-directed mutagenesis

Site-directed mutagenesis was performed on the wild-type vector – ADAMTS13 cDNA containing a carboxy-terminal V5 tag subcloned into a pcDNA4 plasmid (a gift from Dr. J. Evan Sadler, Washington University, St. Louis) – using the Stratagene QuikChange® II XL Site-Directed Mutagenesis Kit (La Jolla, CA).

### Transient transfection

HEK293 cells were transiently transfected with ADAMTS13 plasmid DNA in order to highly express the ADAMTS13 protein. Lipofectamine 2000 by Invitrogen was used to transfect the cells, following the manufacture protocol. Six hours post-transduction, the medium was replaced with Opti-MEM (Invitrogen) medium and cells were maintained in this medium for 24 h.

### VWF collagen ELISA assay

Von Willebrand Factor (VWF) stock solution was prepared by dissolving 1000 IU (CSL Behring, Marburg, Germany) in 20 ml PBS. This stock was served in all the experiments of this study. Five hundred µl of VWF from the stock solution was added to collagen coated wells (LifeDiagnostics *von Willebrand Factor Antigen Assay*, Frenchs Forest, Australia). After three washes the primary antibodies were added for 1 hour incubation (BL154G (Bethyl laboratories, Montgomery, TX, USA), Wh2-22-1A and Wh2-11-1 against ADAMTS13; M0616, A0082 and AXL205 (Dako Corporation, Carpintaria, CA, USA) against VWF and anti-V5 (Invitrogen) against the V5 tag) followed by additional washes and secondary antibody incubation, again for 1 hour. The plate was read at 450 nm. Results were analyzed using Soft Max Pro and Thermo Max microplate reader (Molecular Devices, Sunnyvale, CA, USA).

### Detection of ADAMTS13 protein by flow cytometry

Following trypsinization, cells were washed with PBS, and fixative reagent (IntraPrep^TM^ Beckman Coulter, Marseille, France) (100 µl per each 5×10^5^ cells) was added to the pellet. The cells were vortexed and incubated at room temperature for 15 minutes. After the incubation period, the cells were washed again with PBS for 5 minutes at 1400 RPM in 27°C and the supernatant was aspirated. Permeabilization reagent (IntraPrep^TM^ Beckman Coulter) (100 µl per 5×10^5^ cells) was added to the cell pellet and incubated at room temperature for 5 minutes. Following permeabilization, PBS was added to the solution in order to distribute the cells for the various staining procedures and treatments. Cells were incubated with VWF (0, 10, 100, 250, and 500 µl from VWF stock) after the primary antibody was added (at various temperatures – as described in the Results section for each experiment). Other cells were incubated with the substrate (the same concentrations) before incubation with the primary antibody, and the rest of the cells were incubated with VWF without a primary antibody and only received a secondary antibody, thus serving as controls for non-specific binding in the absence or presence of VWF. In addition, we employed isotopic antibody control (Anti-mouse IgG2a, Invitrogen) to ensure the specificity of the ADAMTS13 antibodies. The concentrations of the primary and secondary antibodies were kept constant through all the treatments. Several dilutions from stock of 1 mg/ml of the monoclonal Wh2-11-1, monoclonal Wh2-22-1A and polyclonal BL154G (Bethyl) antibodies or 1∶100 dilution from 1 mg/ml anti-V5 antibody (Invitrogen) were added to cells and cells were incubated at 37°C for 30 minutes. In some cases, cells were pre-incubated with VWF substrate for 10 minutes at 37°C. Alexa-488 Goat anti-Mouse IgG secondary antibody (Invitrogen) at 37°C for 30 minutes was used for anti-V5, Wh2-11-1 and Wh2-22-1A antibodies while Rabbit anti-Goat IgG (Bethyl) was used for the primary ADAMTS13 antibody BL154G. Certain amounts of VWF were added again to each tube following the addition of the respective specific secondary antibodies. Between adding the primary antibody and the secondary and after adding the secondary antibody, cells were washed with PBS (Invitrogen) 0.1% bovine serum albumin (BSA; Sigma) for 5 minutes and the supernatant was aspirated. The cells were then analyzed using the Becton Dickinson FACSCalibur. Median values were calculated using the “stat” program of CellQuest by Becton Dickinson and were then plotted in Excel. We have recently reported the development and validation of the assay to detect intracellular ADAMTS13 by flow cytometry as well as the detailed protocols [45].

### Preparation of cell lysate and concentrated medium

All experiments were performed on endogenous and recombinant ADAMTS13-expressing cells. We used HEK293 cells, ADAMTS13-HEK293 transduced cells and LX2 stellate liver cells. Twenty-four hours after the transfection, media was collected and concentrated 24–fold using 30 kDa cut-off Centriprep concentrating vials (Millipore, Billerica, MA, USA). The concentrated media was then kept at -20°C. The cells were washed with 5 ml of chilled PBS (Invitrogen) lysed with lysis buffer (1 M Tris-HCl, 5 M NaCl, 100% Triton X-100, Protease Inhibitor Cocktail, PMSF) and then stored at -20°C. A Bradford protein assay (Bio-Rad, New York, NY) was used to determine the total protein concentration for these lysates and concentrated media samples according to the manufacture's protocol.

### Immunoblotting

The samples were boiled with SDSPAGE running buffer for 5 minutes, sonicated for 10 minutes and electrophoresed on 3–8% NUPAGE Tris-Acetate SDS-PAGE gels (Invitrogen). The proteins were then transferred to a nitrocellulose membrane at constant current. The ADAMTS13 protein was detected using the Wh2-11-1 monoclonal primary antibody and anti-mouse HRP secondary antibody (Invitrogen).

### FRET-VWF73 assay

FRET-VWF73 (Peptides International, Louisville, KY, USA) is a flourogenic substrate developed to test the activity of ADAMTS13 [24]. The activity of the protein was tested using a GeminiMax plate reader. Readings were taken every 5 minutes for one hour at a 340 nm excitation and 450 nm emission. The FRET-VWF73 peptide was prepared as a stock concentration of 100 µM according to the manufacturer's instructions and was stored at −20°C. The stock was diluted 25 fold in reaction buffer containing 5 mM Tris-HCl, 25 mM CaCl_2_, and 0.005% Tween-20 (Sigma) at pH 6. The test sample was prepared in a 1∶3 dilution with reaction buffer and 3% NaCitrate solution (Sigma). The FRET-VWF73 and sample dilutions were combined just prior to the kinetic assay. Analysis of the results revealed the rate of ADAMTS13 activity as change in fluorescence per unit protein per minute.

### Statistical analysis

A two-tailed unpaired T-test was used to compare the sample means of immunostaining results at the same concentration levels of antibody. It tests the null hypothesis that two normally distributed independent random samples have equal means. In the homoscedastic case, the test statistic is computed as:
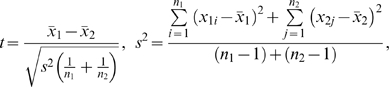



Where 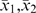 are the sample means, 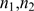 are the sample sizes, and 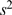 is the unbiased estimate of the variance. The p-values 0.001 are statistically significant at α = 0.05 level.
